# Rescue of expression and function of long QT syndrome-causing mutant hERG channels by enhancing channel stability in the plasma membrane

**DOI:** 10.1016/j.jbc.2024.107526

**Published:** 2024-07-02

**Authors:** Jordan Davis, James D. Cornwell, Noah Campagna, Jun Guo, Wentao Li, Tonghua Yang, Tingzhong Wang, Shetuan Zhang

**Affiliations:** Department of Biomedical and Molecular Sciences, Queen’s University, Kingston, Ontario, Canada

**Keywords:** congenital long QT syndrome, hERG potassium channel, electrophysiology, molecular biology

## Abstract

The *human ether-a-go-go-related gene* (*hERG*) encodes the Kv11.1 (or hERG) channel that conducts the rapidly activating delayed rectifier potassium current (I_Kr_). Naturally occurring mutations in hERG impair the channel function and cause long QT syndrome type 2. Many missense hERG mutations lead to a lack of channel expression on the cell surface, representing a major mechanism for the loss-of-function of mutant channels. While it is generally thought that a trafficking defect underlies the lack of channel expression on the cell surface, in the present study, we demonstrate that the trafficking defective mutant hERG G601S can reach the plasma membrane but is unstable and quickly degrades, which is akin to WT hERG channels under low K^+^ conditions. We previously showed that serine (S) residue at 624 in the innermost position of the selectivity filter of hERG is involved in hERG membrane stability such that substitution of serine 624 with threonine (S624T) enhances hERG stability and renders hERG insensitive to low K^+^ culture. Here, we report that the intragenic addition of S624T substitution to trafficking defective hERG mutants G601S, N470D, and P596R led to a complete rescue of the function of these otherwise loss-of-function mutant channels to a level similar to the WT channel, representing the most effective rescue means for the function of mutant hERG channels. These findings not only provide novel insights into hERG mutation-mediated channel dysfunction but also point to the critical role of S624 in hERG stability on the plasma membrane.

The *human ether-a-go-go-related gene* (*hERG or KCNH2*) encodes the Kv11.1 (or hERG) channel that conducts the rapidly activating delayed rectifier potassium current (I_Kr_), which is important for the repolarization of the cardiac action potential (1). A reduction in I_Kr_ can cause long QT syndrome (LQTS), which predisposes affected individuals to a high risk of fatal cardiac arrhythmias and sudden death (2, 3). Hundreds of loss-of-function hERG mutations have been linked to long QT syndrome type 2 (LQT2) (4), accounting for 30 to 45% of all congenital LQTS in humans (5, 6, 7). Mechanistically, mutations can damage hERG function through various means, including inhibiting protein synthesis due to abnormal transcription/translation, defective protein trafficking, abnormal channel gating, and altered channel permeability (4, 8, 9, 10, 11). While some hERG mutations are nonsense or frame-shift which can lead to mRNA decay (9), a large number of hERG mutations are missense. Most missense mutations decrease hERG expression in the plasma membrane and have been classified as trafficking defective mutant channels (4, 8, 10, 11, 12). Studies have shown that the function of certain trafficking defective mutant channels, including the well-studied hERG mutant G601S, can be rescued by reduced temperature (27 °C) culture (4, 10, 13, 14). However, molecular mechanisms underlying low-temperature rescue are not well understood (4, 10).

hERG channel density in the plasma membrane is maintained through a balance between insertion of newly synthesized channels and degradation of existing channels (15). In the present study, we show that reduced temperature (27 °C) culture slowed the degradation rate, suggesting an enhanced stability, of rescued mature G601S hERG channels. We previously found that, unlike other voltage-gated potassium channels such as Kv1.5, the hERG channel is intrinsically unstable at the plasma membrane when cells are exposed to a reduced extracellular K^+^ concentration (low K^+^) (16). Serine (S) residue at 624 in the innermost position of the selectivity filter of hERG is distinctive as most other K^+^ channels possess threonine (T) residue at the equivalent position (17). We have further found that substitution of serine 624 with threonine (S624T) (herein named “S624T substitution” to distinguish it from LQT2-causing “mutations”) enhances hERG stability and renders hERG insensitive to low K^+^ culture (18). In the present study, we propose that certain mutant hERG channels, such as G601S, may display enhanced instability of the channel, causing it to behave like WT hERG in low K^+^ culture conditions. In other words, the mutant channels may be able to traffic to the plasma membrane but quickly internalize due to instability in the plasma membrane. Since the S624T substitution restores WT hERG stability in low K^+^ conditions (18), here, we intragenically added the S624T substitution to the G601S mutant channel. Our results demonstrated that the intragenic addition of S624T rescued G601S membrane expression and current to a level similar to WT channels. The expression and function of other trafficking defective (loss-of-function) hERG mutants N470D and P596R, but not A561T and Y611H, were also rescued by S624T substitution to a level similar to WT channels. These findings demonstrate the most effective rescue of certain trafficking defective hERG mutants to date and provide insight into the mechanisms that underlie the loss-of-function phenotype of LQTS-causing hERG mutants.

## Results

### Reduced temperature culture rescues the membrane expression and function of G601S hERG mutant

**Figure 1:**
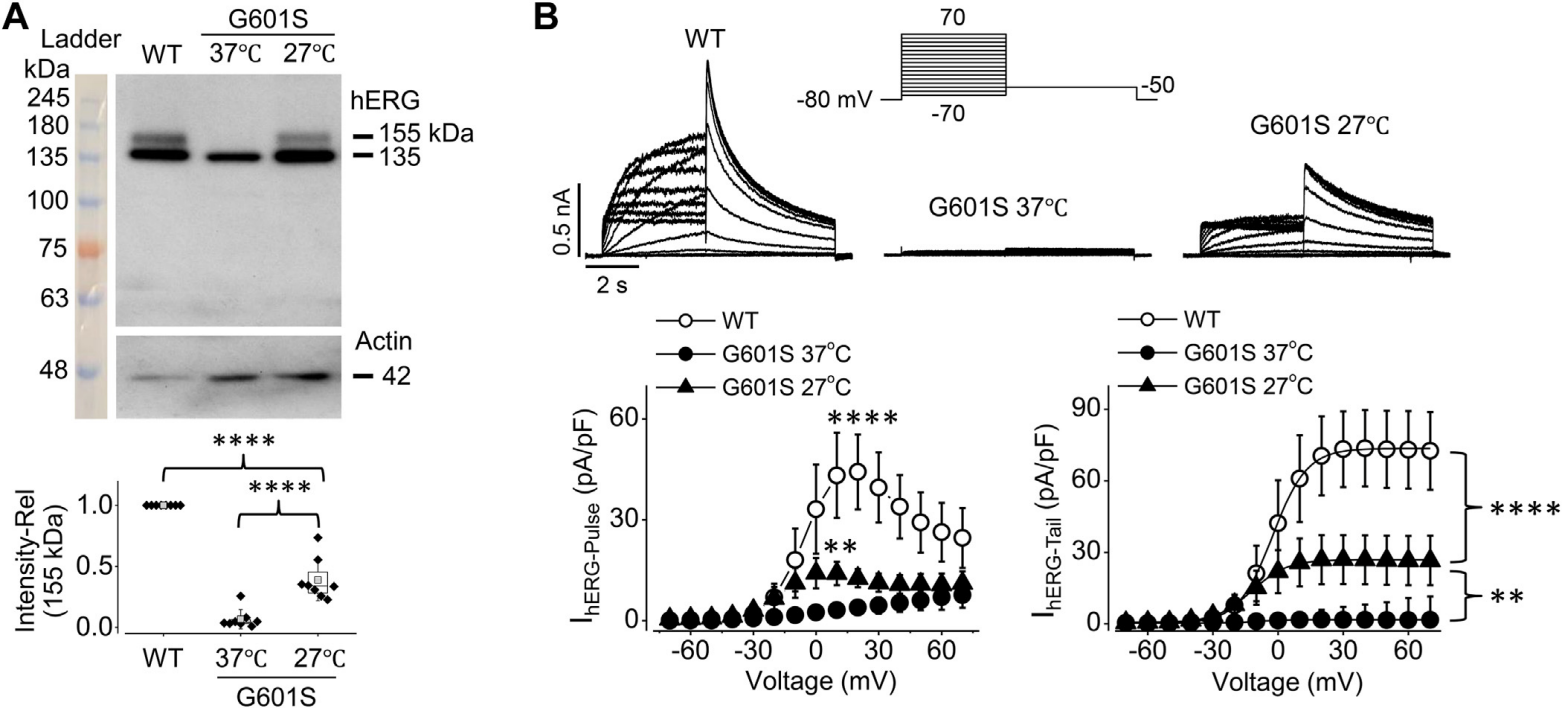
**Reduced temperature (27 °C) culture rescues expression and function of G601S mutant hERG channels.***A*, expression of hERG proteins in HEK cells stably expressing WT hERG as well as G601S mutant hERG cultured at 37 °C or 27 °C for 24 h. Intensities of 155-kDa hERG bands were normalized to their respective actin loading controls then to WT and summarized as box plots with mean ± SD beneath a representative Western blot image (n = 8). ∗∗∗∗*p* < 0.0001 between indicated groups. A one-way ANOVA with Tukey’s post hoc test was used. *B,* families of hERG currents elicited by the voltage protocol shown above in HEK cells stably expressing WT hERG as well as G601S hERG cultured at 37 °C or 27 °C for 24 h. The pulse (I_hERG-Pulse_) and tail (I_hERG-Tail_) currents were summarized from cells in 4 to 6 independent experiments beneath representative current traces (n = 15 for WT hERG, n = 12 for G601S at 37 °C, and n = 13 for G601S at 27 °C). ∗∗*p* < 0.01; ∗∗∗∗*p* < 0.0001 for voltage at 10 mV for pulse currents, and for voltages ≥10 mV for tail currents between G601S at 27 °C and at 37 °C as well as between WT and G601S at 27 °C. ANOVA with Tukey’s post hoc test was used. *Error bars* in B represent SD. HEK, human embryonic kidney; *hERG*, human ether-g-go-go-related gene.

It has been established that hERG proteins from hERG-expressing human embryonic kidney (hERG-HEK) cells under normal culture (37 °C) display two distinct bands with molecular masses of 135 and 155 kDa upon Western blot analysis. The 155-kDa band reflects the mature, fully glycosylated channels localized in the plasma membrane, and the 135-kDa band reflects the immature, core-glycosylated hERG channels localized intracellularly (8, 19, 20, 21, 22, 23, 24, 25, 26). As previously reported (10, 14, 27), in contrast to WT hERG channels, G601S mutant hERG channels expressed in HEK (G601S-HEK) cells displayed the 135-kDa immature band with the absence of 155-kDa mature band (Fig. 1*A*). Consistent with previous studies (4, 27), culturing G601S-HEK cells at reduced temperature (27 °C) for 24 h resulted in the presence of the 155-kDa mature band (Fig. 1*A*).

The rescued mature G601S channels are functional. I_hERG_ from G601S-HEK cells cultured under normal conditions (37 °C) was essentially absent. However, it was present from cells cultured at 27 °C and was less than half the amplitude of I_hERG_ from WT hERG-HEK cells (Fig. 1*B*).

To compare the current-voltage relationships between the rescued G601S and WT hERG currents, we constructed their pulse current (I_hERG-Pulse_)-voltage relationships (Fig. 1*B*, bottom left). Like WT hERG, I_hERG-Pulse_ of rescued G601S was largest around 10 mV and displayed inward rectification upon stronger depolarizing voltages. We also constructed the activation-voltage (g-V) relationships by plotting tail currents (I_hERG-Tail_) upon the −50 mV repolarization step against the depolarization voltages and fitting the plotted data to the Boltzmann function (Fig. 1*B*, bottom right). The half-activation voltages (V_1/2_) and slope factors were −2.7 ± 4.7 mV and 7.2 ± 0.7 mV (n = 15) for WT hERG and −8.6 ± 6.6 mV and 7.2 ± 0.9 mV (n = 13) for rescued G601S. Since tail currents reflect maximal current amplitudes of WT as well as rescued G601S hERG, the peak tail current at −50 mV following a full channel activation (the 50-mV depolarizing step) was used for analyzing current amplitudes.

### Reduced temperature culture decreases the forward trafficking rate and delays the degradation of hERG channels

**Figure 2:**
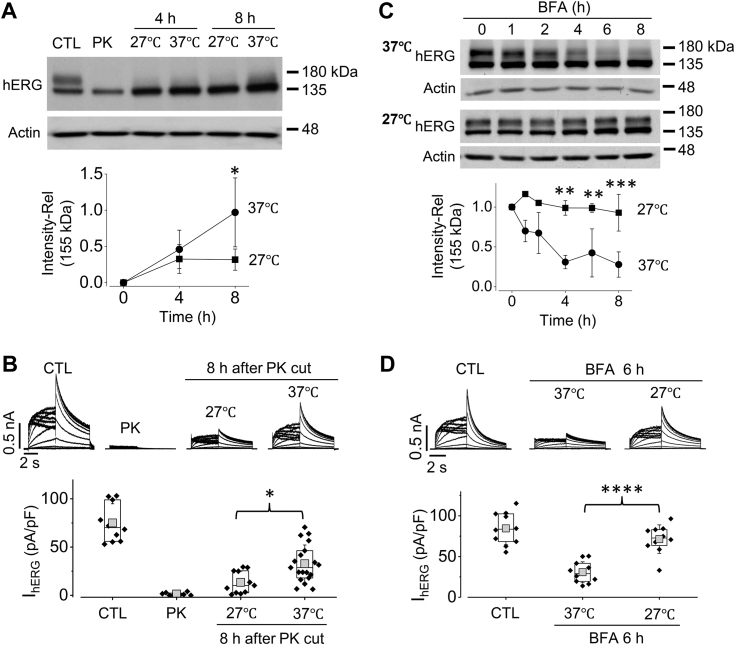
**Reduced temperature (27 °C) culture slows both forward trafficking and degradation of mature hERG channels.***A* and *B*, effects of reduced temperature culture on hERG forward trafficking. WT hERG-HEK cells were treated with proteinase K (PK, 200 μg/ml, 20 min) to completely remove the 155-kDa hERG protein and thus I_hERG_. After the treatment, cells were cultured at 27 °C or 37 °C and collected at different time points for Western blot analysis (*A*) and whole-cell patch clamp recording (*B*). ∗*p* < 0.05 between 27 °C and 37 °C, ANOVA with Tukey’s post hoc test was used. *C* and *D*, effects of reduced temperature culture on mature hERG degradation. hERG-HEK cells were cultured in the presence of BFA (10 μM) under 37 °C or 27 °C and collected at different time points for Western blot analysis (*C*) and whole-cell patch clamp recording (*D*). ∗∗*p* < 0.01; *∗∗∗p* < 0.001; *∗∗∗∗p* < 0.0001 between 27 °C and 37 °C. ANOVA with Tukey’s post hoc test was used. For hERG expression in *A* and *C*, densities of the 155-kDa bands relative to control (CTL, or 0 h) were plotted against time (n = 4). For I_hERG_ in *B* and *D*, peak tail currents from cells in three independent experiments are summarized as box plots with mean ± SD beneath representative current traces. *Error bars* in *A* and *C* represent SD. BFA, brefeldin A; HEK, human embryonic kidney; *hERG*, human ether-g-go-go-related gene.

hERG channel density on the cell surface is determined by both forward trafficking and retrograde degradation (16). Thus, reduced temperature (27 °C) culture-mediated rescue of the mature form (155-kDa) of G601S channels may be *via* either enhancing forward trafficking or impeding retrograde degradation. To this end, we first evaluated the effects of reduced temperature on forward trafficking of WT hERG channels. We cleared existing cell-surface mature (155 kDa) hERG channels of a HEK cell line stably expressing WT-hERG (WT-hERG-HEK) with proteinase K (PK) treatment (8, 20, 22, 24). After removal of the cell surface hERG channels, the hERG-HEK cells were cultured at either 27 °C or 37 °C for various periods. The rate of appearance of newly formed 155-kDa hERG protein represents the forward trafficking rate. Our results revealed that, after the clearance of the 155-kDa protein and I_hERG_ with PK treatment, the recovery rates of 155-kDa hERG expression (Fig. 2*A*) and I_hERG_ (Fig. 2*B*) were slower in 27 °C culture than 37 °C culture. Thus, the forward trafficking rate of hERG was not enhanced, but slowed, by reduced temperature culture.

To understand how reduced temperature affects retrograde degradation of hERG channels, we first treated WT-hERG-HEK cells with brefeldin A (BFA, 10 μM) to inhibit the transport of newly synthesized proteins from the endoplasmic reticulum to the Golgi apparatus, and thus block the conversion of hERG channels from the immature (135 kDa) form to the mature (155 kDa) form (16, 28). In the presence of BFA, the gradual disappearance of the 155-kDa band reflects the natural degradation of the existing mature hERG channels in the plasma membrane. Our results showed that the degradation rates of cell-surface mature hERG protein (155 kDa) (Fig. 2*C*) and I_hERG_ (Fig. 2*D*) were slower in cells under reduced temperature (27 °C) culture than those under normal temperature (37 °C) culture. In other words, reduced temperature culture increases the stability of mature WT hERG channels in the plasma membrane.

### Reduced temperature culture slows the degradation of both WT hERG in 0 K^+^ medium and G601S hERG in normal medium

**Figure 3:**
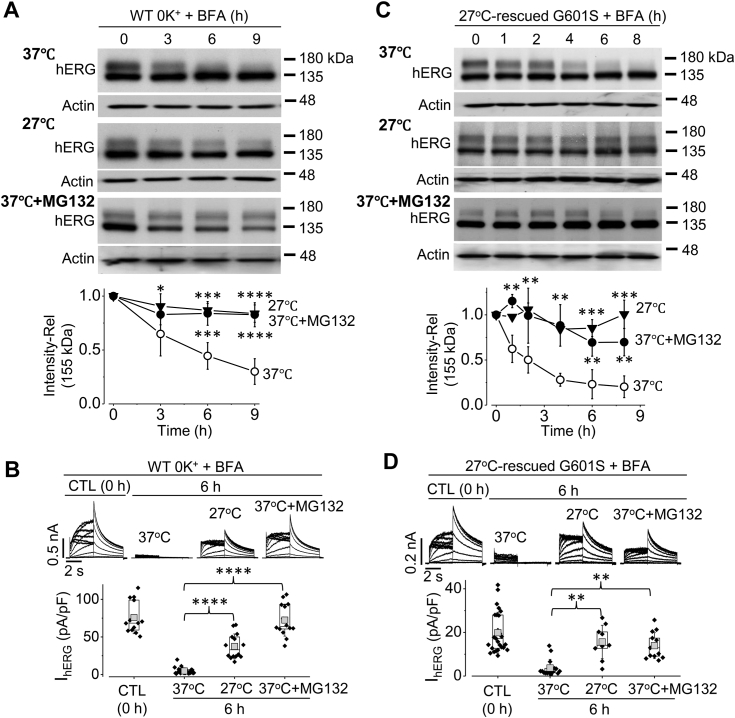
**Reduced temperature (27 °C) culture slows degradation rate of WT hERG in 0 mM K**^**+**^_**o**_**medium and prerescued G601S hERG in normal culture conditions.***A* and *B*, reduced temperature culture or MG132 (10 μM) slows the degradation rate of WT hERG in 0 mM K^+^_o_ medium. hERG-HEK cells were cultured with BFA (10 μM) in 0 mM K^+^ medium at 37 °C, 27 °C, or 37 °C + MG132 for various periods. After culture, cells were collected for Western blot analysis (*A*) and whole-cell patch clamp recordings (*B*). ∗*p* < 0.05; ∗∗∗*p* < 0.001; ∗∗∗∗*p* < 0.0001 between 37 °C and 27 °C or 37 °C + MG132. ANOVA with Tukey’s post hoc test was used. *C* and *D*, reduced temperature culture or MG132 (10 μM) slows the degradation rate of pre-rescued G601S hERG in normal culture medium. G601S hERG cells were cultured at 27 °C for 24 h to restore the 155-kDa expression. The cells were then cultured with BFA (10 μM) in normal medium at 37 °C, 27 °C, or 37 °C + MG132 for various periods. After culture, cells were collected for Western blot analysis (*C*) and whole-cell patch clamp recordings (*D*). ∗∗*p* < 0.01; ∗∗∗*p* < 0.001 between 37 °C and 27 °C or 37 °C + MG132. ANOVA with Tukey’s post hoc test was used. For Western blot analyses in *A* and *C*, relative 155-kDa band intensities were normalized to their respective actin loading controls then to the initial value (0 h) in each gel, and data are summarized beneath representative Western blot images (n = 3–6). For I_hERG_ in *B* and *D*, peak tail currents from cells in 3 to 4 independent experiments are summarized as box plots with mean ± SD beneath representative current traces. *Error bars* in *A* and *C* represent SD. BFA, brefeldin A; HEK, human embryonic kidney; *hERG*, human ether-g-go-go-related gene.

We previously demonstrated that extracellular K^+^ (K^+^_o_) is a prerequisite for WT hERG channels to remain stable in the plasma membrane. In normal culture medium (5 mM K^+^), WT hERG channels in the plasma membrane maintain a relatively stable density. In the absence (0 mM) of K^+^ in the culture medium, WT hERG channels in the plasma membrane are unstable and completely degrade within a few hours (16, 29). To study whether reduced temperature affects 0 mM K^+^ induced degradation of WT hERG channels, we treated hERG-HEK cells with BFA (10 μM) to eliminate the potential interference from forward trafficking of newly synthesized mature (155-kDa) hERG channels. We then cultured cells in 0 mM K^+^ medium at 37 °C, 27 °C, or 37 °C with the proteasomal inhibitor MG132 (10 μM) for various periods. WT hERG expression and I_hERG_ were examined using Western blot analysis and whole-cell voltage clamp recordings. Compared to normal temperature (37 °C) culture, reduced temperature (27 °C) culture, or MG132 treatment slowed the 0 mM K^+^ culture-induced degradation of the mature (155 kDa) proteins (Fig. 3*A*) and reduction in I_hERG_ (Fig. 3*B*) of WT channels.

To investigate whether reduced temperature also slows the degradation of G601S hERG channels on the membrane, we first rescued mature hERG proteins (155 kDa) of G601S channels by culturing G601S-HEK cells at 27 °C for 24 h. We then treated cells with BFA (10 μM) to exclude the potential interference from forward trafficking and cultured cells at either 37 °C, 27 °C, or 37 °C with the proteasomal inhibitor MG132 (10 μM) for various periods. The rescued mature hERG protein (155 kDa) of G601S channels under normal temperature (37 °C) and normal K^+^ (5 mM) culture was unstable and underwent rapid degradation. Reduced temperature (27 °C) or MG132 treatment slowed the time-dependent degradation of the mature protein (155 kDa) (Fig. 3*C*) and reduction in I_hERG_ of G601S hERG channels (Fig. 3*D*).

Thus, reduced temperature culture slowed the degradation of both WT hERG in 0 mM K^+^_o_ and rescued G601S in normal K^+^ culture. These results raised the possibility that the G601S in normal K^+^ culture may be able to reach the plasma membrane but behave like WT hERG in 0 mM K^+^_o_ culture conditions; the mature channels of G601S are unstable in the plasma membrane and degrade, leading to the lack of the mature (155 kDa) channel protein and the current.

**Figure 4:**
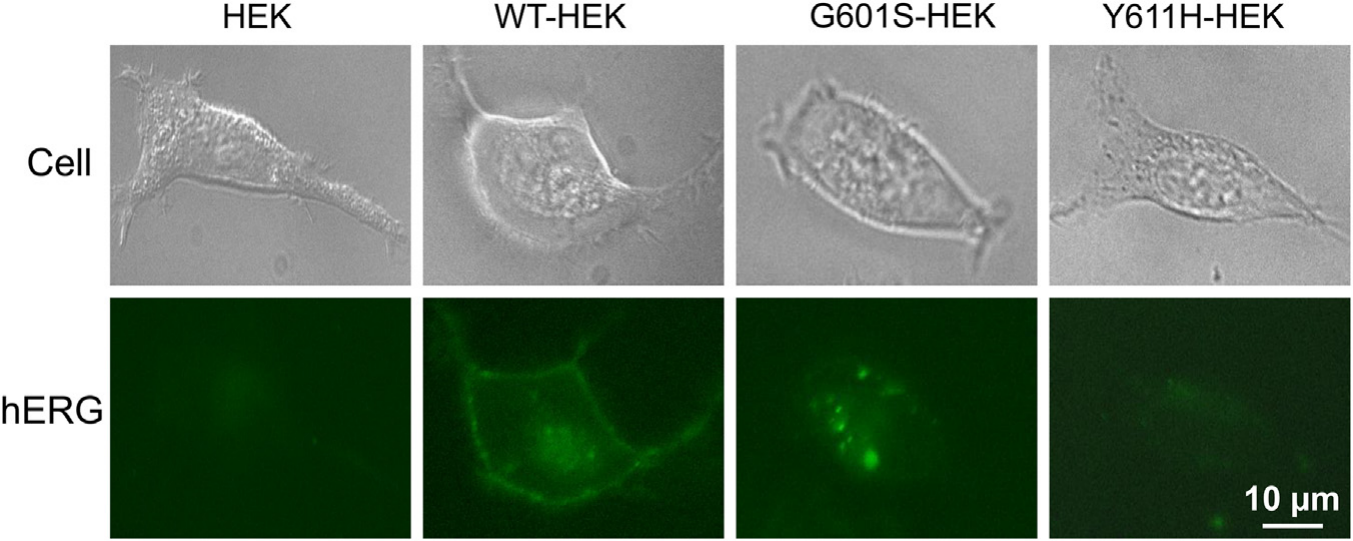
**G601S hERG channels can be labelled with an antibody that only targets cell-surface hERG channels.** Control HEK cells and HEK cells stably expressing WT, G601S, or Y611H were cultured for 30 min in medium containing FITC-conjugated anti-hERG antibody that binds to an epitope in the extracellularly exposed S1-S2 linker of hERG channels. After washing unbound antibodies from the medium, live-cell images were taken using an inverted fluorescence microscope. The scale bar represents 10 μm. HEK, human embryonic kidney; *hERG*, human ether-g-go-go-related gene.

To directly address this possibility, we cultured HEK cells (negative control) and HEK cells stably expressing WT, G601S (that can be rescued by reduced temperature-culture) or Y611H (that cannot be rescued by reduced temperature-culture) (4, 10) hERG channels for 30 min in medium containing FITC-conjugated anti-hERG antibody that targets an epitope in the extracellularly exposed S1-S2 linker of hERG channels. Since the antibody used does not infiltrate cell membranes (30), FITC staining only occurs for cell-surface localized mature hERG channels. After washing out unbound hERG antibody from the medium, fluorescent images were taken. The untransfected HEK cells displayed no signal, indicating that the hERG antibody is specific and cannot enter live cells. The WT hERG-expressing cells displayed both plasma membrane and intracellular signals, the G601S-expressing cells primarily displayed punctate intracellular signals, and Y611H-expressing cells did not show meaningful signals. Thus, WT hERG mature channels remain mostly on the cell surface while some are internalized. Whereas Y611H hERG cannot reach the membrane and thus were not labeled, mature G601S hERG channels can reach the cell membrane where they are labeled by the antibody but are unstable and subsequently internalized (Fig. 4). These data suggest that instability of certain mutant hERG channels, such as G601S, contributes to the diminished membrane expression.

### Intragenic addition of S624T substitution rescues expression and function of the G601S mutant

**Figure 5:**
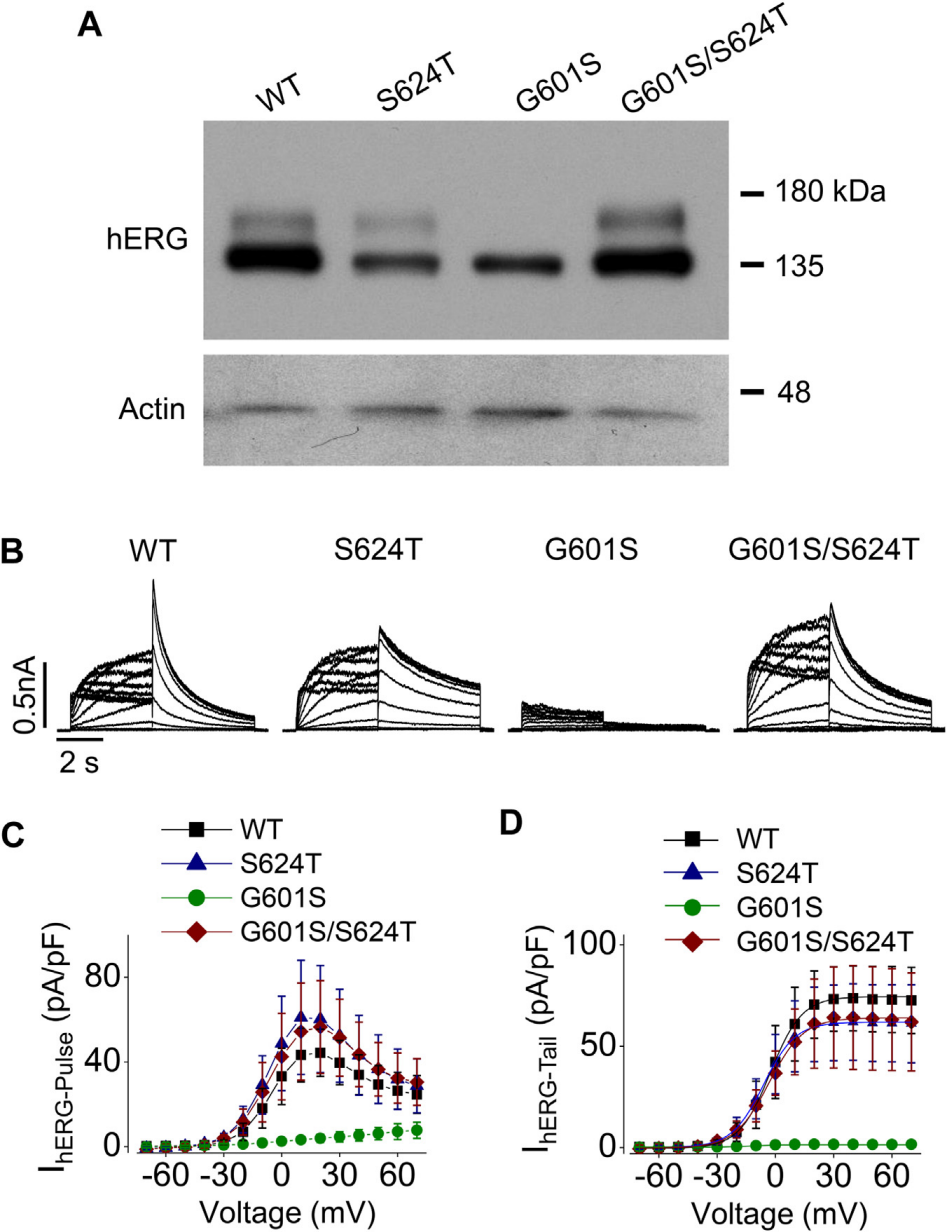
**Intragenic addition of S624T mutation rescues the membrane expression and current of the G601S hERG channel.***A*, representative Western blot images of whole-cell lysate from HEK cells stably expressing WT, S624T, G601S, or G601S/S624T hERG channels (n = 7). *B*, families of I_hERG_ traces elicited by the voltage protocol shown in Figure 1*B* from HEK cells stably expressing WT, S624T, G601S, or G601S/S624T hERG channels. *C* and *D*, summarized I_hERG-Pulse_- and I_hERG-Tail_-voltage relationships of these channels from cells in three independent experiments (n = 12 for WT, n = 9 for S624T, n = 12 for G601S, and n = 9 for G601S/S624T). *Error bars* in C and D represent SD. HEK, human embryonic kidney; *hERG*, human ether-g-go-go-related gene.

Through culturing hERG-HEK cells in 0 mM K^+^ medium, we previously demonstrated that WT hERG channels are intrinsically unstable in the plasma membrane under hypokalemic conditions and are completely internalized within 6 h when cells are exposed to 0 mM K^+^ culture media (16). By systematically mutating individual amino acid residues of the pore region, we identified that substituting S624 with T (S624T) can enhance hERG stability, such that after cells are exposed to 0 mM K^+^ medium for 12 h, S624T hERG channels remain on the cell surface and are functional (18). Thus, we identified a site (*e.g.*, S624) that is critical for hERG membrane stability such that the S624T substitution enhances the stability of mature hERG channels in the plasma membrane. To enhance the membrane stability of G601S, we added the S624T substitution into the G601S mutant channel to construct a mutant hERG channel containing both G601S and S624T (G601S/S624T-hERG), and then we created a HEK cell line stably expressing G601S/S624T-hERG mutant (G601S/S624T-HEK cells). As shown in Figure 5, the addition of S624T intragenically rescued the mature channel expression and the channel function. In contrast to G601S-HEK cells, G601S/S624T-HEK cells cultured at normal temperature (37 °C) showed robust mature (155-kDa) hERG protein expression (Fig. 5*A*). Whole-cell patch clamp recordings demonstrated a robust current of G601S/S624T with an amplitude similar to that of WT hERG (Fig. 5*B*). Specifically, while I_hERG_ of G601S was 1.6 ± 1.2 pA/pF (n = 12), I_hERG_ of G601S/S624T was 63.7 ± 9.6 pA/pF (n = 9). I_hERG_ was 73.17 ± 4.7 pA/pF (n = 12) for WT, and 61.4 ± 6.3 pA/pF (n = 9) for S624T hERG channels. The currents at the end of each depolarizing step were plotted against the depolarizing voltages to construct pulse current-voltage relationships. Similar to WT hERG channels, G601S/S624T and S624T displayed characteristic bell-shaped pulse current-voltage relationships (Fig. 5*C*). The tail currents upon −50 mV repolarization were plotted against the depolarizing voltages to construct the activation-voltage relationships, which were fitted to the Boltzmann equation to obtain the half-activation voltages (V_1/2_) and slope factors for each channel (Fig. 5*D*). The V_1/2_ and slope factors were −2.7 ± 4.7 mV and 7.2 ± 0.7 mV (n = 12) for WT hERG, −6.8 ± 5.6 mV and 7.7 ± 0.9 mV (n = 9) for S624T, and −4.6 ± 6.1 mV and 7.9 ± 0.7 mV (n = 9) for G601S/S624T.

### Intragenic addition of S624T substitution rescues N470D and P596R, but not A561T or Y611H mutant hERG channels

**Figure 6:**
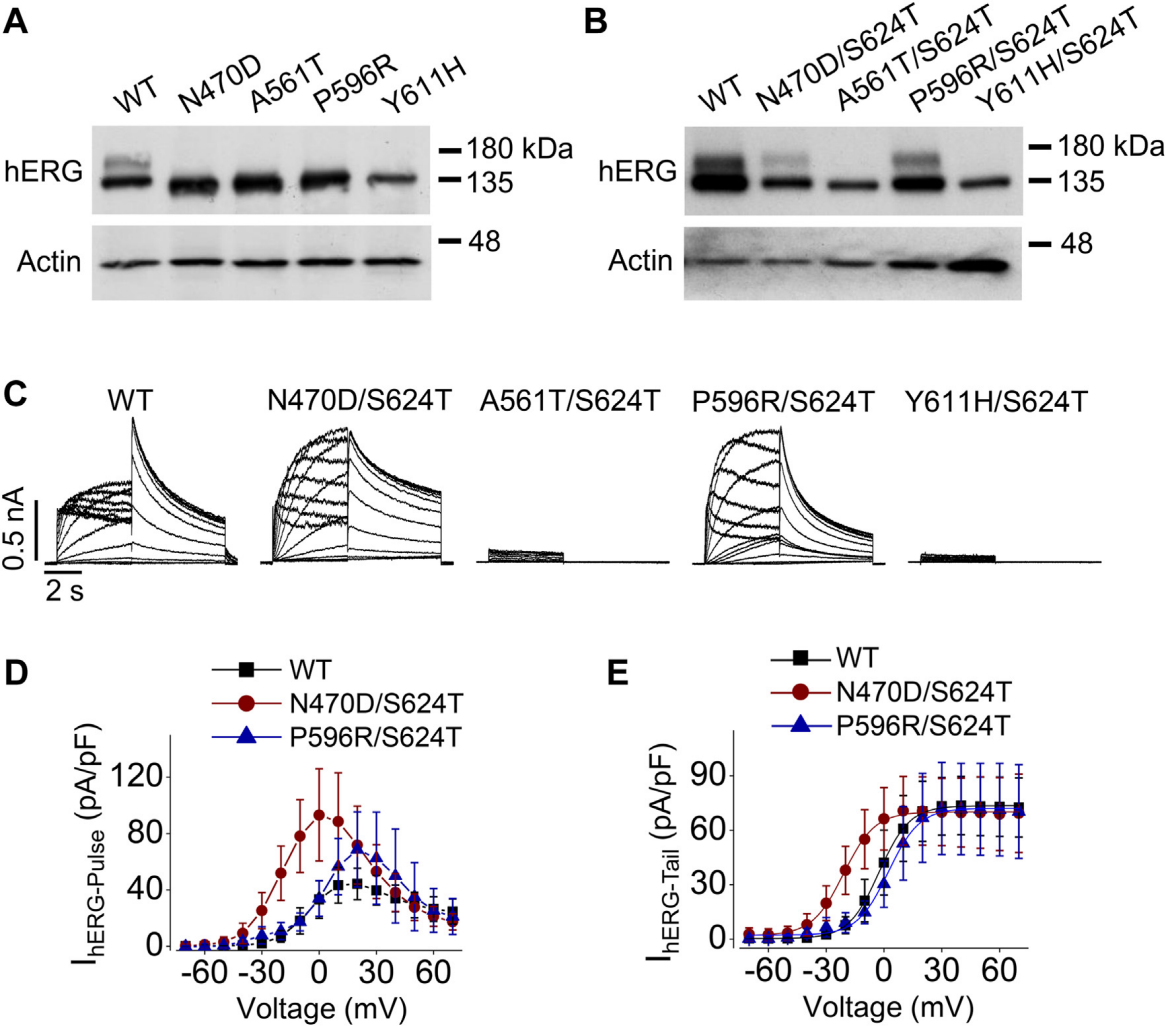
**Intragenic addition of S624T substitution rescues the membrane expression and current of N470D and P596R, but not A561T or Y611H mutant hERG channels**. *A*, Western blot images of hERG expression from whole-cell lysate of HEK cells expressing WT, N470D, A561T, P596R, and Y611H hERG channels (n = 5). *B*, Western blot images of hERG expression from whole-cell lysate of HEK cells stably expressing WT, N470D/S624T, A561T/S624T, P596R/S624T, or Y611H/S624T hERG channels (n = 9). *C*, families of I_hERG_ traces elicited by the voltage protocol shown in Figure 1*B* from HEK cells stably expressing WT, N470D/S624T, A561T/S624T, P596R/S624T, or Y611H/S624T hERG channels. *D* and *E*, summarized I_hERG-Pulse_- and I_hERG-Tail_-voltage relationships from cells in three to four independent experiments (n = 12 for WT, n = 10 for N470D/S624T, n = 10 for P596R/S624T). *Error bars* in *D* and *E* represent SD. HEK, human embryonic kidney; *hERG*, human ether-g-go-go-related gene.

Since the expression of G601S hERG mutant was rescued by the S624T substitution, we wondered whether other loss-of-function hERG mutants could also be rescued by S624T substitution. To this end, S624T was added to LQT2-causing hERG mutants N470D, A561T, P596R, or Y611H. These mutants were chosen because they are also loss of function and are believed to be due to trafficking defects. As shown in Figure 6*A*, upon Western blot analysis, different from WT hERG channels which displayed both the 135-kDa (immature) and the 155-kDa (mature) bands, N470D, A561T, P596R, and Y611H hERG mutants only displayed the 135-kDa band. The addition of S624T substitution rescued the 155-kDa band for N470D and P596R, but not for A561T or Y611H hERG mutants (Fig. 6*B*). Whole-cell patch clamp analysis also demonstrated that addition of S624T substitution rescued the function of N470D and P596R, but not of A561T or Y611H mutants (Fig. 6*C*). Similar to WT hERG, both N470D/S624T and P596R/S624T displayed bell-shaped pulse current-voltage relationships (Fig. 6*D*). The activation-voltage relationships were constructed by plotting tail currents against activation voltages and fitted to Boltzmann function. The V_1/2_ and slope factors were −2.7 ± 4.7 mV and 7.2 ± 0.7 mV (n = 12) for WT hERG; −21.0 ± 7.1 mV and 7.3 ± 1.2 mV (n = 10) for N470D/S624T; and 2.5 ± 5.7 mV and 7.3 ± 1.5 mV (n = 10) for P596R/S624T (Fig. 6*E*). Thus, compared to WT hERG, the half-activation voltage of N470D/S624T was shifted to the negative direction by ∼20 mV (*p* < 0.0001).

Interestingly, the S624T-mediated rescue occurs in mutant hERG channels that can be rescued by reduced temperature culture. Specifically, while the function of N470D and P596R can be rescued by reduced temperature culture, A561T and Y611H cannot (10, 31). Furthermore, the S624T-rescued mutant channel kinetics are similar to those rescued by reduced temperature. For example, current of N470D channels rescued by reduced temperature culture or hERG blocking agents also displayed a negative shift of the V_1/2_ compared to that of WT hERG channels (31, 32).

### Intragenic addition of hERG mutation G601S, N470D, or P596R makes S624T sensitive to 0 mM K^+^ culture

**Figure 7:**
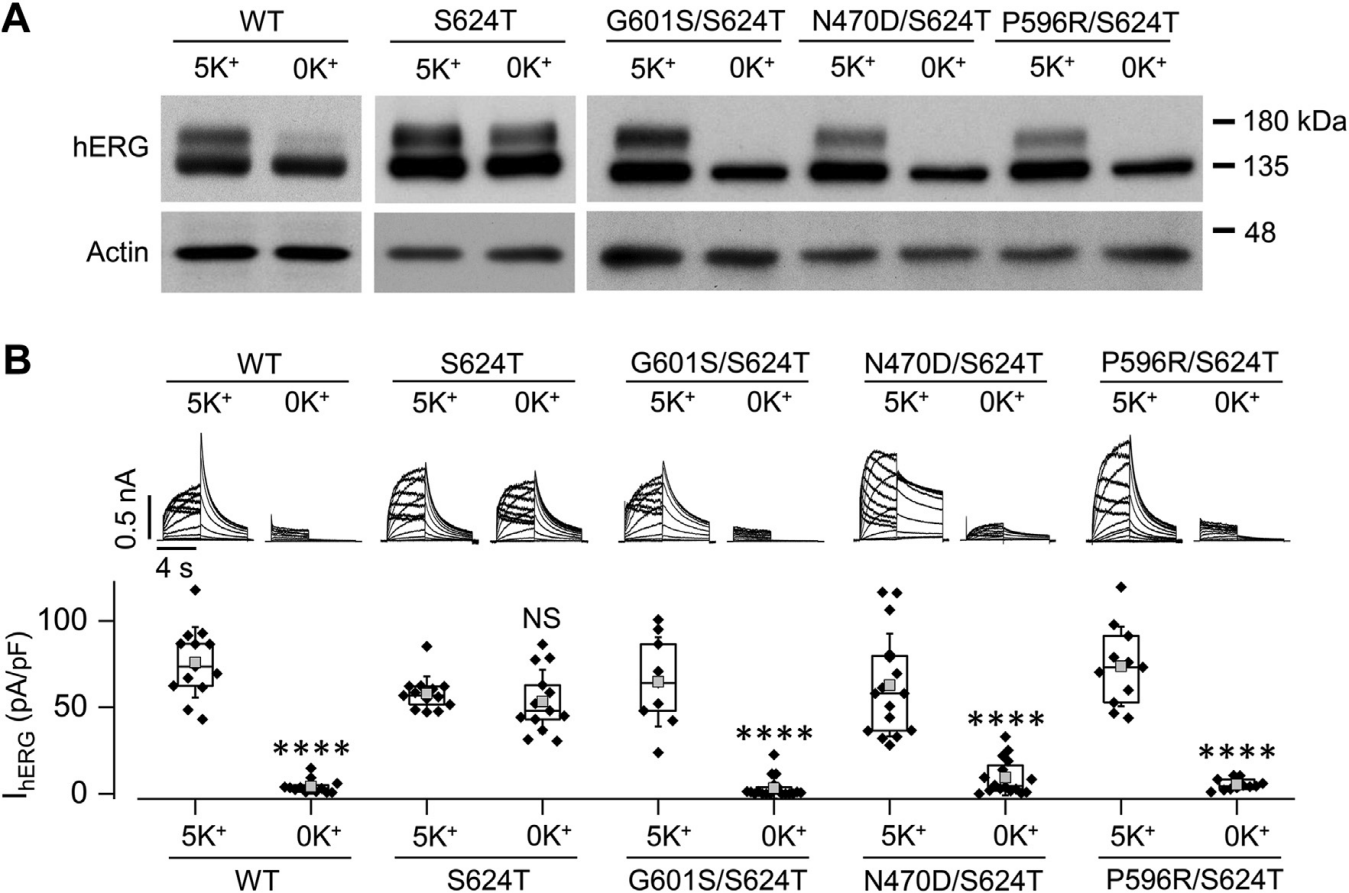
**Intragenic addition of the “trafficking-defective” mutation G601S, N470D, or P596R makes S624T sensitive to 0 mM K**^**+**^**exposure.** HEK cells stably expressing WT, S624T, G601S/S624T, N470D/S624T, or P596R/S624T hERG channels were cultured in either 5 mM K^+^ or 0 mM K^+^ medium for 6 h. After the treatment, cells were collected for Western blot analysis and whole-cell voltage clamp recordings. *A*, representative Western blot images (n = 3–5). *B*, peak tail current from cells in three to five independent experiments are summarized as box plots with mean ± SD beneath representative current traces. NS, not significant; ∗∗∗∗*p* < 0.0001 compared to 5 mM K^+^ medium. A two-tailed unpaired Student’s *t* test was used. HEK, human embryonic kidney; *hERG*, human ether-g-go-go-related gene.

We have shown that S624T hERG is more stable than WT hERG channels in low K^+^_o_ conditions; in contrast to WT hERG channels whose mature channels degrade in 0 mM K^+^ culture, S624T hERG channels display both mature and immature channels in 0 mM K^+^ culture (18). To directly demonstrate hERG mutations can induce membrane instability of the channel, we examined channel expression and activity in response to 0 mM K^+^ medium culture when various hERG mutants were added to S624T. HEK cells stably expressing WT, S624T, G601S/S624T, N470D/S624T, or P596R/S624T hERG channels were cultured with 5 mM or 0 mM K^+^ media for 6 h and then examined with Western blot analysis (Fig. 7*A*) and whole-cell patch clamp recordings (Fig. 7*B*). As expected (18), mature hERG (155-kDa) expression and I_hERG_ of WT hERG, but not of S624T hERG, were reduced in 0 mM K^+^ culture compared to those in 5 mM K^+^ culture. Interestingly, G601S/S624T, N470D/S624T, and P596R/S624T all displayed sensitivity to 0 mM K^+^ culture. The mature protein (155 kDa) expression and I_hERG_ were reduced following 6-h culture in 0 mM K^+^ medium. Thus, intragenic addition of hERG mutations G601S, N470D, or P596R rendered S624T hERG sensitive to 0 mM K^+^ induced endocytic degradation.

## Discussion

hERG/I_Kr_ plays an important role in the repolarization of human cardiac action potentials (2, 3). Many naturally occurring missense hERG mutations, such as G601S (13), disrupt protein expression at the plasma membrane, leading to a loss of function of the channel, causing LQT2 (4). This group of mutations have been classified as trafficking defective mutant hERG channels (10). The new findings of our present study demonstrate that some of the trafficking defective mutant hERG channels, such as G601S, can traffic to the plasma membrane, but are unstable, resulting in a lack of cell surface expression and loss-of-function. Enhancing membrane stability of these mutant channels by reduced temperature culture or the intragenic substitution of S624T can effectively rescue their plasma membrane expression and function.

It is well reported that reduced temperature culture can rescue the plasma membrane expression and function of many trafficking defective mutant hERG channels (4, 10, 31, 33), however, the underlying mechanisms are not fully understood. It has been suggested that mutant hERG channels, such as G601S, are rescued by reduced temperatures due to an increase in forward trafficking from the ER, where it is usually sequestered at normal culture temperature (27). Newly synthesized hERG is processed in the ER, where core glycosylation occurs, and then it is taken to the Golgi apparatus to undergo complex glycosylation before being sent to the plasma membrane (26, 34). During this process, hERG interacts with multiple chaperones and cochaperones including HSP 70 and HSP 90. There is evidence that reduced temperature culture can modulate the affinity between hERG and HSP 70 or HSP 90, which represents a possible explanation for the temperature dependence of folding and forward trafficking of hERG (35). On the other hand, membrane stability and endocytic degradation contribute to the density of hERG channels on the plasma membrane (15). It has been reported that reduced temperature culture inhibits energy-dependent endocytic processes (36). One study reported that cell-surface expression of hERG was higher at reduced culture temperature (37); reduced temperature inhibited intracellular endosomal transport following endocytosis, as well as endocytosis itself (37). In the present study, our results show that reduced temperature (27 °C) culture rescued G601S hERG plasma membrane expression and function (Fig. 1). Moreover, our results for the first time demonstrate that reduced temperature (27 °C) culture did not accelerate but slowed forward trafficking of hERG channels and delayed the degradation of mature hERG channels (Fig. 2). These results indicate that enhanced stability of mature channels at the plasma membrane is likely to be responsible for reduced temperature culture-mediated rescue of G601S expression and function.

We previously demonstrated that cell surface WT hERG channels are unstable and undergo complete endocytic degradation when cells are exposed to 0 mM K^+^ medium (16). The 0 mM K^+^_o_ culture-induced internalization of WT hERG mirrored the internalization of prerescued G601S at physiological culture conditions (5 mM K^+^, 37 °C), and reduced temperature culture slowed the degradation of both WT hERG in 0 mM K^+^ medium culture and prerescued G601S mutant hERG channels in normal culture conditions (Fig. 3). Our immunocytochemical data indicate that G601S, but not Y611H, hERG channels can reach the plasma membrane but are unstable and quickly internalize (Fig. 4), suggesting reduced temperature culture may rescue certain trafficking defective mutant hERG channels by stabilizing their presence in the plasma membrane. We previously demonstrated that the S624T substitution stabilizes hERG channels in the plasma membrane, enabling mature channels to maintain at the cell surface in 0 mM K^+^ medium (18). Most intriguingly, our present study demonstrates that the addition of S624T to G601S (G601S/S624T) intragenically rescued plasma membrane expression and function of G601S channels (Fig. 5). The S624T substitution also rescued trafficking defective hERG mutants N470D and P596R, but not A561T and Y611H (Fig. 6). In a previous study that used intragenic mutations to rescue mutant hERG channels, Delisle *et al.* demonstrated that the Y652C substitution and to a lesser extent the Y652S substitution partially rescued expression and function of G601S hERG channels (38). In the present study, the intragenic S624T substitution rescued the currents of mutant hERG channels (G601S, N470D, and P596R) to a level that is comparable to WT hERG channels (Figs. 5 and 6), representing the most effective rescue to date. These findings also indicate that S624 plays a vital role in the stability of hERG channels. It is noted that S624T substitution-mediated rescue only occurred in mutants that can also be rescued by reduced temperature culture (*e.g.*, G601S, N470D, or P596R), but not those that cannot be rescued by reduced temperature culture (*e.g.*, A561T or Y611H) (4, 10). These observations suggest that membrane-instability plays a key role in the loss of function of certain previously thought trafficking defective mutant hERG channels (*e.g.*, G601S, N470D, and P596R), which can be rescued by reduced temperature culture or S624T substitution *via* stabilizing the channels in the plasma membrane.

Due to its distinct structure, the hERG channel possesses unique flexibility (17). For example, most K^+^ channels have a GYG signature amino acid sequence for K^+^ selectivity filter and two tryptophans (WW) at the N-terminal end of the pore-loop. It is thought that the nitrogen atoms of the WW and the hydroxyls of Y may form hydrogen bonds, contributing to the rigidity of the channels (39). However, hERG has a selectivity filter with a GFG sequence, and the corresponding WW are replaced by YF. As well, hERG has a uniquely long extracellular S5-pore linker (17, 24) and a large internal pore cavity due to the lack of the Pro-X-Pro sequence, which exists in the S6 domain of other K^+^ channels and causes a sharp bend to reduces the internal pore mouth (17, 40, 41). As a result, hERG is more flexible compared to other K^+^ channels, and extracellular K^+^ binding is required to maintain its structural stability and function (18). S624 lies at the base of the pore helix in hERG, adjacent to the GFG selectivity filter. Substitution of serine with threonine (S624T) makes the channel more rigid in the plasma membrane (18); while WT hERG loses its conductance in minutes and loses its plasma membrane expression in hours when cells are exposed to 0 mM K^+^ medium, S624T hERG is resistant to 0 mM K^+^ medium (18). This provides a mechanistic explanation for how S624T imparted stability on G601S, N470D, and P596R at the plasma membrane.

The overall flexibility of the hERG channel would be expected to depend on more complex and delicate amino acid residue interactions, and therefore certain mutations like G601S, N470D, or P596R may decrease the channel stability. This notion is directly supported by our results obtained using low K^+^ medium culture; while S624T enhances hERG stability and makes the channel resistant to 0 mM K^+^ medium, introducing the mutation G601S, N470D, or P596R to S624T (*e.g.,* G601S/S624T, N470D/S624T, or P596R/S624T) makes the channel sensitive to 0 mM K^+^_o_ culture-induced degradation (Fig. 7). Thus, these mutations impart enough instability onto S624T so that S624T can no longer withstand the destabilizing effect induced by exposure to 0 mM K^+^_o_ medium.

It is known that hERG blockers can rescue the mature channel expression on plasma membrane of some trafficking defective mutant hERG channels, but the underlying molecular mechanisms are not well understood (28, 31, 42, 43). Since S624 in hERG is involved in the binding of many hERG blockers (41, 44, 45), drug binding to S624 may produce an effect similar to S624T substitution, stabilizing hERG channels in the plasma membrane. It would be interesting for future research to identify compounds that preferentially target S624 without markedly blocking the channel to rescue mutant hERG channels.

In summary, while it is generally thought trafficking defective mutant hERG channels retain inside the cell, our data in the present study suggests that some of these channels may reach the plasma membrane and membrane instability contributes to the lack of their cell surface expression and function. Reduced temperature may rescue these mutant hERG channels by stabilizing their membrane expression. The fact that the intragenic addition of the more membrane stable S624T substitution can robustly rescue the function of hERG mutants, such as G601S, N470D, and P596R, that can also be rescued by reduced temperature supports our notion. Our data also indicate the involvement of the amino acid residue at the innermost position (*i.e.*, 624) of the channel selectivity filter in hERG membrane stability. These findings provide new insights into mutation-mediated channel dysfunction, which has implications for strategies of rescuing LQTS-causing mutant hERG channels.

## Experimental procedures

### Molecular biology

*hERG* cDNA was provided by Dr Gail Robertson (University of Wisconsin-Madison). The hERG S624T, G601S, N470D, A561T, P596R, and Y611H point mutations as well as G601S/S624T, N470D/S624T, A561T/S624T, P596R/S624T, and Y611H/S624T double mutations were generated using PCR (*PfuUltra* Hotstart PCR Master Mix, Agilent Technologies) and confirmed by DNA sequencing (Azenta Life Sciences). Lipofectamine 2000 (Invitrogen) was used for transfecting plasmids into HEK cells. Stable cell lines were generated using G418 for selection (1 mg/ml) and maintenance (0.4 mg/ml). HEK cells were cultured in minimum essential medium (Invitrogen) supplemented with 10% fetal bovine serum (Invitrogen), 1 mM sodium pyruvate, and nonessential amino acids (Thermo Fisher Scientific). For transient expression, 24 hours after transfection, cells were collected for biochemical and patch clamp experiments.

### Patch clamp recording

The whole-cell patch clamp method was used to record hERG currents (I_hERG_). Cells collected from plates after culture were allowed to settle on the bottom of the recording chamber perfused with the bath solution. Patch glass pipettes were pulled from thin-walled borosilicate glass (World Precision Instruments,). The inner diameter of pipettes was approximately 1.5 μm with a resistance of about 2 MΩ when filled with solution. An Axopatch 200B amplifier and pCLAMP10 (Molecular Devices) were used for data acquisition and analysis. Series resistance was compensated by 80%. Leak subtraction was not used. The pipette solution consisted of 135 mM KCl, 5 mM MgATP, 5 mM EGTA, and 10 mM HEPES (pH 7.2 with KOH). The bath solution contained 135 mM NaCl, 5 mM KCl, 2 mM CaCl_2_, 1 mM MgCl_2_, 10 mM glucose, and 10 mM HEPES (pH 7.4 with NaOH). I_hERG_ was evoked by depolarizing cell membrane to voltages between −70 and 70 mV with 10-mV increments. The tail currents were recorded upon a repolarizing step to −50 mV. The holding potential was −80 mV. For the current amplitude analyses, the peak tail current at −50 mV following the 50-mV depolarizing step was used. Patch clamp experiments were performed at room temperature (22 ± 1 °C).

### Western blot analysis

Whole-cell proteins were isolated and Western blot analysis was performed using the previously described procedure (16, 18, 46). Proteins were separated on 8% SDS-PAGE, transferred onto a polyvinylidene difluoride membrane, and blocked for 1 h with 5% nonfat milk. The blots were incubated with the primary antibody for 1 h at room temperature and then incubated with a horseradish peroxidase (HRP)-conjugated secondary antibody. Actin expression was used for loading controls. The blots were visualized with X-ray film (Fujifilm, Minato) using the enhanced chemiluminescence detection kit (Cytiva). Molecular masses of proteins were determined using BLUeye prestained protein ladder (GeneDireX). Image Lab software (Bio-Rad) was used to quantify band intensities. To obtain relative expression values, band intensities in various conditions were normalized to their respective actin intensities and then enumerated relative to their corresponding control.

### Immunofluorescence microscopy

HEK cells without transfection or HEK cells stably expressing WT, G601S, or Y611H hERG were transferred and cultured for 24 h in 35 mm glass-bottom dishes (World Precision Instruments). Then, cells were cultured in minimum essential medium with 3% bovine serum albumin and FITC-conjugated anti-Kv11.1 antibody that targets an epitope in the extracellularly exposed S1-S2 linker of hERG (Sigma-Aldrich) at a concentration of 1:25 at 37 °C for 30 min. Afterward, the cells were washed three times with PBS to remove unbound antibodies. Images were taken with a ZEISS Axio Observer Z1 inverted fluorescence microscope.

### Reagents and antibodies

Goat anti-hERG (C-20, N-20) and mouse anti-hERG (F-12) primary antibodies and mouse anti-goat IgG-HRP—conjugated secondary antibodies were obtained from Santa Cruz Biotechnology. Goat anti-rabbit and horse anti-mouse IgG-HRP conjugated secondary antibodies were obtained from Cell Signaling. Rabbit anti-Kv11.1 (hERG), FITC-conjugated anti-Kv11.1, and mouse anti-actin (AC-40) antibodies, G418, PK, and BFA were purchased from Sigma-Aldrich.

### Statistical analysis

All data are expressed as the mean ± SD. For scattered plots, the means (gray square) ± SD (open square) are overlapped with original data points. For experiments with multiple groups, a one-way ANOVA with Tukey’s post hoc test was used; for experiments between two groups, a two-tailed unpaired Student’s *t* test was used for statistical significance. A *p* value of 0.05 or less was considered significant.

## Data availability

All data are contained within the manuscript.

## Conflict of interest

The authors declare that they have no conflicts of interest with the contents of this article.
